# Social Determinants of Health Phenotypes and Cardiometabolic Condition Prevalence Among Patients in a Large Academic Health System: Latent Class Analysis

**DOI:** 10.2196/53371

**Published:** 2024-08-07

**Authors:** Carrie R Howell, Li Zhang, Olivio J Clay, Gareth Dutton, Trudi Horton, Michael J Mugavero, Andrea L Cherrington

**Affiliations:** 1 Division of Preventive Medicine, Department of Medicine, University of Alabama at Birmingham, Birmingham, AL, United States; 2 School of Public Health, University of Alabama at Birmingham, Birmingham, AL, United States; 3 Alzheimer’s Disease Research Center, University of Alabama at Birmingham, Birmingham, AL, United States; 4 Deep South Resource Center for Minority Aging Research, University of Alabama at Birmingham, Birmingham, AL, United States; 5 Department of Psychology, University of Alabama at Birmingham, Birmingham, AL, United States; 6 Division of Infectious Diseases, Department of Medicine, University of Alabama at Birmingham, Birmingham, AL, United States

**Keywords:** social determinants of health, electronic medical record, phenotypes, diabetes, obesity, cardiovascular disease, obese, social determinants, social determinant, cardiometabolic, risk factors, risk factor, latent class analysis, cardiometabolic disease, EMR, EHR, electronic medical record, electronic health record

## Abstract

**Background:**

Adverse social determinants of health (SDoH) have been associated with cardiometabolic disease; however, disparities in cardiometabolic outcomes are rarely the result of a single risk factor.

**Objective:**

This study aimed to identify and characterize SDoH phenotypes based on patient-reported and neighborhood-level data from the institutional electronic medical record and evaluate the prevalence of diabetes, obesity, and other cardiometabolic diseases by phenotype status.

**Methods:**

Patient-reported SDoH were collected (January to December 2020) and neighborhood-level social vulnerability, neighborhood socioeconomic status, and rurality were linked via census tract to geocoded patient addresses. Diabetes status was coded in the electronic medical record using *International Classification of Diseases* codes; obesity was defined using measured BMI ≥30 kg/m^2^. Latent class analysis was used to identify clusters of SDoH (eg, phenotypes); we then examined differences in the prevalence of cardiometabolic conditions based on phenotype status using prevalence ratios (PRs).

**Results:**

Complete data were available for analysis for 2380 patients (mean age 53, SD 16 years; n=1405, 59% female; n=1198, 50% non-White). Roughly 8% (n=179) reported housing insecurity, 30% (n=710) reported resource needs (food, health care, or utilities), and 49% (n=1158) lived in a high-vulnerability census tract. We identified 3 patient SDoH phenotypes: (1) high social risk, defined largely by self-reported SDoH (n=217, 9%); (2) adverse neighborhood SDoH (n=1353, 56%), defined largely by adverse neighborhood-level measures; and (3) low social risk (n=810, 34%), defined as low individual- and neighborhood-level risks. Patients with an adverse neighborhood SDoH phenotype had higher prevalence of diagnosed type 2 diabetes (PR 1.19, 95% CI 1.06‐1.33), hypertension (PR 1.14, 95% CI 1.02‐1.27), peripheral vascular disease (PR 1.46, 95% CI 1.09‐1.97), and heart failure (PR 1.46, 95% CI 1.20‐1.79).

**Conclusions:**

Patients with the adverse neighborhood SDoH phenotype had higher prevalence of poor cardiometabolic conditions compared to phenotypes determined by individual-level characteristics, suggesting that neighborhood environment plays a role, even if individual measures of socioeconomic status are not suboptimal.

## Introduction

The population prevalence of cardiometabolic disease continues to rise [1], increasing patient burden and societal costs. Cardiometabolic disease disproportionately impacts those with low socioeconomic status (SES) [1-4], with marked geographic variations in diabetes and obesity prevalence across the United States [3]. Growing evidence suggests that social determinants of health (SDoH) [5] influence cardiometabolic disease.

SDoH refer to the social, economic, and environmental conditions in which people are born, live, and work and how these factors influence their health and well-being. These determinants include factors such as SES, education, employment, housing, access to health care, and the broader social and community context. Prior studies have reported that individuals with adverse SDoH, such as those with lower overall SES [6,7] or who live in underresourced neighborhoods [8], are more likely to have cardiometabolic disease. SDoH exacerbate patient burden and contribute to rising cardiometabolic-related conditions. Innovative ways to approach cardiometabolic prevention and management that consider both clinical and SDoH measures at the population level are needed to address cardiometabolic outcomes and related disparities.

One innovative approach is to use SDoH data from the electronic medical record (EMR) to identify those who are at highest risk for cardiometabolic disease or who may need more focused clinical management. Currently, social risk or SDoH screening in health care settings is rapidly gaining steam and has been advocated by multiple academies, health profession organizations, and the US Centers for Medicare and Medicaid Services [9-13]. Standardized tools that accurately and thoroughly capture relevant SDoH in the EMR are emerging. One of the commonly used tools—the Protocol for Responding to and Assessing Patient Assets, Risks, and Experience (PRAPARE) tool [14,15]—queries patients about social risks such as individual SES, housing, food insecurity, and stress as well as current residential address in order to geocode and link in neighborhood-level data such as census tract indicators (eg, percentage in tract living in poverty, employed, and educated). Despite the PRAPARE being one of the most commonly used tools in practice, there is little published literature on how best to operationalize and act upon patient responses. A few studies have reported on the implementation of the PRAPARE [15-17], with recent work from our group highlighting the range of social risks that patients in our health system catchment experience [18].

Disparities in poor cardiometabolic outcomes are rarely a result of a single risk factor—whether it is at the individual or neighborhood level. For instance, the number of adverse SDoH (eg, count) have been associated with increased risk of coronary heart disease mortality [19] and incident stroke [20]. Beyond SDoH count, grouping individuals based on shared combinations of factors could provide valuable information for tailored health prevention approaches [21,22]. For instance, investigators [22] identified clusters of SDoH in women living with HIV and found a distinct cluster of women that experienced discrimination and stigma along with economic hardship who were at increased risk of recent drug use, providing a distinct high-risk subpopulation suitable for tailored interventions. Defining SDoH phenotypes associated with poor health outcomes, including diabetes disparities [3], to tailor health care delivery is advocated in recent literature [23,24].

Clustering can be accomplished using mixture modeling such as latent class analysis. This approach uses multiple indicators to identify homogeneous subgroups—or phenotypes—with similar characteristics within a heterogeneous population [25]; these phenotypes usually have distinct features that result in divergent outcomes [26,27], such as differential treatment effects by phenotype [28]. While some studies have clustered social determinants in general [29], neighborhood-level determinants [30], obesity-related health behaviors [31-33], clinical factors associated with cardiometabolic disease [34,35], and most recently SDoH, in the All of Us study [29], no study to date has identified SDoH phenotypes for cardiometabolic-related conditions.

Clustering comprehensive SDoH data collected via the EMR at both the individual and neighborhood level may uncover unique patient phenotypes to prioritize intervention and clinical care in diabetes. Therefore, in this study, we aimed to identify and characterize SDoH phenotypes based on patient-reported (eg, PRAPARE) and neighborhood-level data from a large health system’s EMR and evaluate the prevalence of diabetes, obesity, and other cardiometabolic conditions by phenotype status.

## Methods

### Study Population

For this study, we used data from patients who were administered the PRAPARE at the University of Alabama at Birmingham (UAB) Health System. The UAB is located in the Deep South—a geographic and cultural region in the southeastern United States. Alabama, which is at the core of this geographic region, has a higher prevalence of cardiovascular disease and diabetes than the United States overall (8.1% vs 6.4% and 14.8% vs 10.6%, respectively, in 2020 [36]), as well as persistent disparity by SES factors such as education and household income within the state. Jefferson County houses the UAB Hospital system, with approximately 40% of the hospital’s community inpatient discharges per year living in Jefferson County and an additional 35% residing in 29 surrounding counties.

Collection of the PRAPARE was implemented in January 2020 in the ambulatory service at UAB, which included patients who visited a community health or emergency clinic. Information on integrating the PRAPARE and the overall study population have been previously published [18]. Briefly, the PRAPARE was administered to every patient referred to the hospital social work service, resulting in roughly 6500 patients completing at least 1 PRAPARE assessment between January 1 and December 31, 2020. Using these data, we further excluded those who (1) were missing any data on the PRAPARE [18], (2) were missing neighborhood-level data, (3) were younger than 18 years at the time of assessment, and (4) were missing diabetes or obesity status (the main conditions of interest).

### Ethical Considerations

The institutional review board (IRB) of the University of Alabama at Birmingham reviewed and approved this study (IRB-300007801), and the procedures followed in accordance with the ethical standards of the institutional review board and the Helsinki Declaration of 1975. The original consent or IRB approval covers secondary analysis without additional consent.

### Individual-Level SDoH

Individual-level, self-reported SDoH were collected via the PRAPARE. The PRAPARE consists of 21 questions across 4 domains: personal characteristics, family and home, money and resources, and social and emotional health. Answers to each question were categorized and coded for analysis such that higher scores indicated adverse SDoH. Table 1 shows specific items and coding. Items included being afraid of one’s partner, veteran status, housing status, incarceration status, housing insecurity, stress, social support, resource needs (money for rent and utilities), safety where one lives, education level, employment level, access to transportation, and insurance status.

**Table 1.** Characteristics of patients identified in the electronic medical record with social risk data available for latent class analysis (LCA) in 2020 by diabetes status.

			Total (N=2380)	Diabetes (n=894)	No diabetes (n=1486)	*P* value	Coded for LCA
**Personal characteristics**							
	Age (years), mean (SD)		53.1 (16.3)	57.9 (14.0)	50.2 (16.9)	<.001	—^a^
	**Age (years), n (%)**					<.001	—
		<40	537 (22.6)	85 (9.5)	452 (30.4)		
		40-60	973 (40.9)	394 (44.1)	579 (39)		
		≥60	870 (36.5)	415 (46.4)	455 (30.6)		
	**Gender, n (%)**					.78	—
		Male	975 (41)	363 (40.6)	612 (41.2)		
		Female	1405 (59)	531 (59.4)	874 (58.8)		
	**Race, n (%)**					<.001	—
		White	1182 (49.7)	349 (39)	833 (56.1)		
		Black or other	1198 (50.3)	545 (61)	653 (43.9)		
	**Spoken language, n (%)**					.09	
		English	2329 (97.9)	869 (97.2)	1460 (98.3)		1
		Other	51 (2.1)	25 (2.8)	26 (1.7)		2
	**Migrant work in last 2 years, n (%)**					.89	
		No	2366 (99.4)	889 (99.4)	1477 (99.4)		1
		Yes	14 (0.6)	5 (0.6)	9 (0.6)		2
	**Veteran, n (%)**					.007	
		No	2255 (94.7)	833 (93.2)	1422 (95.7)		1
		Yes	125 (5.3)	61 (6.8)	64 (4.3)		2
**Family and home, n (%)**							
	**Housing status**					.008	
		I have housing	2299 (96.6)	875 (97.9)	1424 (95.8)		1
		I do not have housing	81 (3.4)	19 (2.1)	62 (4.2)		2
	**Housing insecurity**					.03	
		Not worried about losing housing	2201 (92.5)	840 (94)	1361 (91.6)		1
		Worried about losing housing	179 (7.5)	54 (6)	125 (8.4)		2
**Socioeconomic status, n (%)**							
	**Education**					.27	
		More than high school	1073 (45.1)	396 (44.3)	677 (45.6)		1
		High school diploma or General Educational Development certificate	1018 (42.8)	377 (42.2)	641 (43.1)		2
		Less than a high school degree	289 (12.1)	121 (13.5)	168 (11.3)		3
	**Employment**					<.001	
		Employed	612 (25.7)	174 (19.5)	438 (29.5)		1
		Retired	997 (41.9)	422 (47.2)	575 (38.7)		2
		Unemployed	771 (32.4)	298 (33.3)	473 (31.8)		3
	**Insurance**					<.001	
		Private	1162 (48.8)	475 (53.1)	687 (46.2)		1
		Public	774 (32.5)	306 (34.2)	468 (31.5)		2
		Self-pay	444 (18.7)	113 (12.6)	331 (22.3)		3
	**Lack of resources**					.69	
		None reported	1670 (70.2)	623 (69.7)	1047 (70.5)		1
		Needed	710 (29.8)	271 (30.3)	439 (29.5)		2
	**Transportation**					.03	
		No need	2135 (89.7)	815 (91.2)	1320 (88.8)		1
		Nonmedical need	154 (6.5)	43 (4.8)	111 (7.5)		2
		Medical need	91 (3.8)	36 (4)	55 (3.7)		3
**Social and emotional health, n (%)**							
	**Stress**					<.001	
		Not at all	574 (24.1)	262 (29.3)	312 (21)		1
		A little bit	589 (24.7)	229 (25.6)	360 (24.2)		2
		Quite a bit	324 (13.6)	115 (12.9)	209 (14.1)		3
		Somewhat	537 (22.6)	190 (21.3)	347 (23.4)		4
		Very much	356 (15)	98 (11)	258 (17.4)		5
	**Social support**					<.001	
		More than 5 times a week	1202 (50.5)	469 (52.5)	733 (49.3)		1
		3 to 5 times a week	804 (33.8)	322 (36)	482 (32.4)		2
		1 or 2 times a week	246 (10.3)	71 (7.9)	175 (11.8)		3
		Less than once a week	128 (5.4)	32 (3.6)	96 (6.5)		4
**Personal safety and vulnerability, n (%)**							
	**Safe where I live**					.09	
		Yes	2231 (93.7)	848 (94.9)	1383 (93.1)		1
		No	149 (6.3)	46 (5.1)	103 (6.9)		2
	**Afraid of my partner**					.001	
		No	2318 (97.4)	883 (98.8)	1435 (96.6)		1
		Yes	62 (2.6)	11 (1.2)	51 (3.4)		2
	**Incarcerated**					<.001	
		No	2320 (97.5)	887 (99.2)	1433 (96.4)		1
		Yes	60 (2.5)	7 (0.8)	53 (3.6)		2
	**Refugee**					.17^b^	
		No	2371 (99.6)	893 (99.9)	1478 (99.5)		1
		Yes	9 (0.4)	1 (0.1)	8 (0.5)		2
**Neighborhood level, n (%)**							
	**Social Vulnerability Index^c^ overall**					.03	
		Low	508 (21.3)	178 (19.9)	330 (22.2)		1
		Moderate	714 (30)	250 (28)	464 (31.2)		2
		High	1158 (48.7)	466 (52.1)	692 (46.6)		3
	**Urbanicity**					.002	
		Metropolitan	2060 (86.6)	798 (89.3)	1262 (84.9)		1
		Micropolitan	188 (7.9)	54 (6)	134 (9)		2
		Small town	88 (3.7)	22 (2.5)	66 (4.4)		3
		Rural	44 (1.8)	20 (2.2)	24 (1.6)		4
	**Yost index^d^**					<.001	
		High	279 (11.7)	103 (11.5)	176 (11.8)		1
		Moderate	734 (30.8)	233 (26.1)	501 (33.7)		2
		Low	1367 (57.4)	558 (62.4)	809 (54.4)		3

a Not applicable (not used in LCA).

b The Fisher exact test was applied.

c Higher values indicate more vulnerabilities in census tract of residence.

d Lower values indicate worse neighborhood-level socioeconomic status in census tract of residence.

### Neighborhood-Level SDoH

The PRAPARE collects residential addresses, which the health system geocodes to the census tract level, giving us the ability to link publicly available neighborhood-level data for each patient at the time of PRAPARE assessment. To globally assess the social environment of a patient’s place of residence, we used the US Centers for Disease Control and Prevention 2018 Social Vulnerability Index (SVI) [37], a composite index that uses 15 census-data indicators of social factors ranked at the tract level across the United States to describe the social conditions that may influence human suffering and financial hardship (ie, social vulnerabilities). To capture the specific socioeconomic environment of place of residence, we used the Yost neighborhood SES index (nSES) [38-43] at the census tract level, which includes 7 components from the census that cover categories of SES including education, income and home values, as well as employment status, and has been used in cancer outcome research as well as integrated into the Surveillance, Epidemiology, and End Results (SEER) registries [44]. Rurality was characterized using the 2010 US Department of Agriculture rural-urban commuting area (RUCA) codes [45]. Additional information on the indices and codes used can be found in Multimedia Appendix 1, Table S1 [40,41].

### Cardiometabolic Conditions

The main cardiometabolic conditions of interest were type 2 diabetes (T2D) and obesity (for those with BMI available), as well as the clinical measure of uncontrolled glycosylated hemoglobin (HbA_1c_). Conditions were extracted from the EMR over the same 12-month time period as the PRAPARE. T2D was defined using *International Classification of Diseases, Tenth Revision* (*ICD-10)* and SNOMED codes extracted from the EMR problem list. BMI was calculated using vital signs (height and weight); BMI ≥30 kg/m^2^ was considered obese. HbA_1c_ (for those with data available) was categorized as uncontrolled at ≥7.0 units. To account for multiple encounters, we used mean clinical values. Other cardiometabolic chronic conditions—hypertension, coronary artery disease, myocardial infarction, peripheral vascular disease, cardiomyopathy, and heart failure—were examined and defined using *ICD-10* and SNOMED codes extracted from the EMR problem list.

### Analysis

We characterized the study population overall and then compared PRAPARE and neighborhood SDoH by diabetes status using the *χ*^2^ and Fisher exact tests as appropriate. To detect SDoH phenotypes in our data, latent class analysis (LCA)—a statistical method that can be used to detect subgroups within populations that share common characteristics—was used [25]. This method uses patterns of responses to observed variables to identify unobserved (or latent) variables in samples [46-48]—such as class membership. It is useful for classifying phenotypes that could benefit from a similar intervention based on their shared characteristics [48,49].

To conduct the LCA, we first coded all SDoH indicator variables so that a higher score indicated risk for that respective category (see Table 1). Out of 16 self-reported variables assessed on the PRAPARE, we included 13 variables in the LCA as well as 3 neighborhood-level SDoH that were linked using address of residence, for a total of 16 variables to define the hypothesized unobserved classes: afraid of one’s partner, veteran, housing status, incarcerated, housing insecurity, stress, social support, resource needs, safety where one lives, education, employment, transportation, insurance status, SVI, rurality, and the Yost SES index. We excluded migrant, refugee, and language status due to low prevalence among participants (<2.5%). We fit a sequence of models using the R (version 4.0.5; R Foundation for Statistical Computing) package *poLCA* and examined multiple fit statistics and interpretability to determine the final model.

Fit statistics included information criteria, with lower values equaling a better fit: (1) the Bayesian information criterion (BIC), which is considered the most reliable indicator of fit; (2) the Akaike information criterion (AIC); (3) sample-size adjusted BIC (aBIC); and (4) consistent AIC (cAIC). We also examined classification diagnostics such as the number of sample members in each class and did not consider models with classes that contained less than 5% of the sample [50]. Entropy, indicating how accurately the model defined classes, was considered, with a value of >0.80 deemed ideal [51].

After determining the best model, the class conditional response probabilities for all variables were estimated by each level, with the estimated mixing proportions corresponding to the share of observations belonging to each latent class. An alternate method for determining the size of the latent classes is to assign each observation to a latent class on an individual basis according to its model posterior class membership probability. Congruence between these 2 sets of population shares often indicates a good fit of the model to the data. We then compared SDoH and neighborhood characteristics by phenotype and calculated prevalence ratios (PRs) to compare differences in cardiometabolic prevalence by phenotype status. Lastly, to determine whether phenotype status differed across various demographic levels, we calculated prevalence odds ratios (PORs) with demographic variables (age, gender, and race) as explanatory variables and phenotype status as the outcome.

## Results

**Figure 1.**
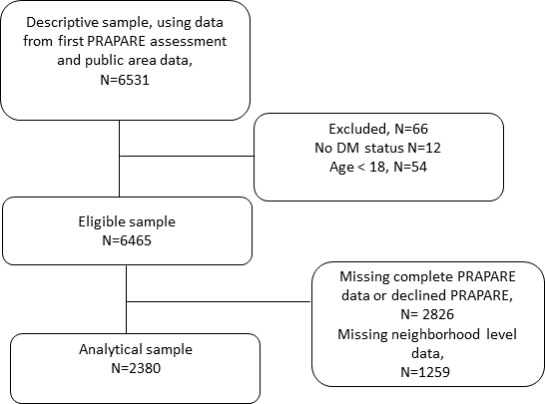
Flow of patients with social risk assessments identified in the institution’s electronic medical record in 2020 to use for clustering analysis. DM: diabetes mellitus. PRAPARE: Protocol for Responding to and Assessing Patient Assets, Risks, and Experience.

The flow of assessments used for clustering can be found in Figure 1. After removing those that were ineligible or missing data, we identified n=2380 for analysis. The characteristics of the analytic sample overall and by diabetes status, as well as how indicator variables were coded, are presented in Table 1. Overall, our sample had a mean age of 53 (SD 16.3) years; 59% (n=1405) were female, 50% were non-White (n=1198), and 38% had a diabetes diagnosis (n=894). In bivariate associations, patients with diabetes were more likely to be non-White, have veteran status, be unemployed or retired, and live in an adverse neighborhood as defined by the SVI, RUCA, and nSES.

**Figure 2.**
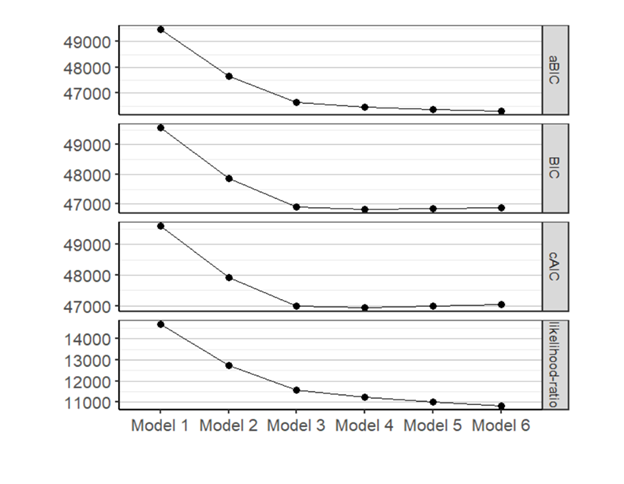
Elbow plot of comparisons of latent class analysis models using patients identified in the electronic medical record with social risk data available in 2020. A significant reduction in criteria is observed in the elbow plot before model 3. The difference between model 3 and model 4 is minimal. aBIC: adjusted Bayesian information criterion; BIC: Bayesian information criterion; cAIC: consistent Akaike information criterion.

Results for the LCA for different class models are presented in Table 2, with the elbow plot presented in Figure 2. The fit statistics, specifically the BIC and cAIC, suggested a 3- or 4-class model, while class size and entropy (divergence of the classes) were considered good for both models. We then considered that, in LCA, large data sets with multiple indicators can result in producing additional classes that lead to a decreased BIC/cAIC since these fit statistics favor more complex models. After examining the elbow plot, we determined that inflection occurred at the 3-class point, with minimal loss of information from the 4-class model; thus, we selected the 3-class model to characterize SDoH phenotypes.

**Figure 3.**
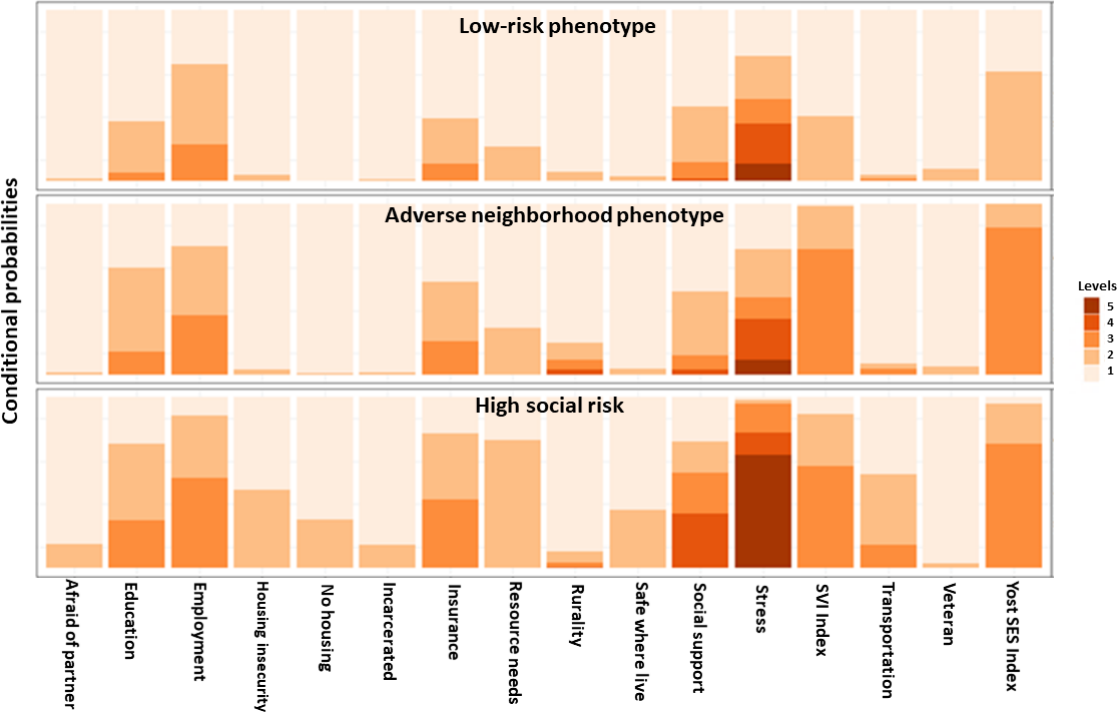
Conditional probabilities by latent class for each variable by cluster type using patients identified in the electronic medical record with social risk data available in 2020. Darker colors indicate a higher proportion of reporting adverse social determinants of health. SES: socioeconomic status; SVI: Social Vulnerability Index.

Figure 3 shows the conditional probabilities by phenotype for each SDoH indicator used in the analysis. Indicator variables were coded with higher scores reflecting adverse SDoH, with the darker columns indicating higher conditional probabilities for the SDoH for patients in that phenotype. After studying the conditional probabilities, we found that the first phenotype (low risk) reported the lowest individual social risks in general and did not predominantly live in adverse neighborhoods. The second phenotype (adverse neighborhood SDoH) tended to live in neighborhoods with higher social vulnerability and lower SES. The third phenotype (high social risk) indicated the highest probabilities of reporting individual social risks compared to the other clusters, as well as moderate levels of living in adverse neighborhoods.

**Table 2.** Fit statistics of latent class analysis models using patients identified in the electronic medical record with social risk data available in 2020.

Model	Log-likelihood (*df*)		AIC^a^	BIC^b^	aBIC^c^	cAIC^d^	Likelihood ratio	Entropy	Smallest class size, %
Model 1	−24,673.87 (2351)		49,405.7	49,573.21	49,481.07	49,602.21	14,692.14	—^e^	—
Model 2	−23,696.81 (2321)		47,511.6	47,852.33	47,664.87	47,911.33	12,738.02	0.879	34
Model 3^f^	−23,110.59 (2291)		46,399.1	*46,913.15*	46,630.38	*47,002.15*	11,565.59	*0.841*	10
Model 4	−22,948.80 (2261)		46,135.6	*46,822.80*	46,444.72	*46,941.80*	11,242.00	*0.738*	10
Model 5	−22,838.17 (2231)		45,974.3	46,834.79	46,361.38	46,983.79	11,020.74	0.715	7
Model 6	−22,739.48 (2201)		45,836.9	46,870.66	46,301.93	47,049.66	10,823.36	0.689	6

a AIC: Akaike information criterion.

b BIC: Bayesian information criterion.

c aBIC: adjusted Bayesian information criterion.

d cAIC: consistent Akaike information criterion.

e Not applicable.

f Model selected; italicized values indicate main criteria used to compare models 3 and 4 to determine best model fit and interpretability.

We then characterized the phenotypes using clinical, demographic, and SDoH data (Table 3). Interestingly, the high social risk phenotype was over 50% male, was younger, had the lowest prevalence of diabetes and—as shown in the conditional probabilities plot—had a higher prevalence of lacking housing, having less than a high school education, being unemployed, reporting self-payer status, and lacking resources and transportation, as well as a high level of stress and moderately higher level of living in an adverse neighborhood. Alternatively, patients classified into the adverse neighborhood phenotype were older, non-White, and lived in census tracts with higher vulnerability and lower SES, as well as rural and small-town locales.

When we examined differences in the prevalence of cardiometabolic conditions by phenotype status (Table 4), we found that the prevalence of diabetes (PR 1.19, 95% CI 1.06‐1.33), hypertension (PR 1.14, 95% CI 1.02‐1.27), peripheral vascular disease (PR 1.46, 95% CI 1.09‐1.97), and heart failure (PR 1.46, 95% CI 1.20‐1.79) was greater among those with an adverse neighborhood phenotype compared to patients with the low-risk phenotype. Surprisingly, patients with a high social risk phenotype did not have higher prevalence of diabetes, obesity, or cardiovascular outcomes compared to those in the low-risk phenotype.

**Figure 4.**
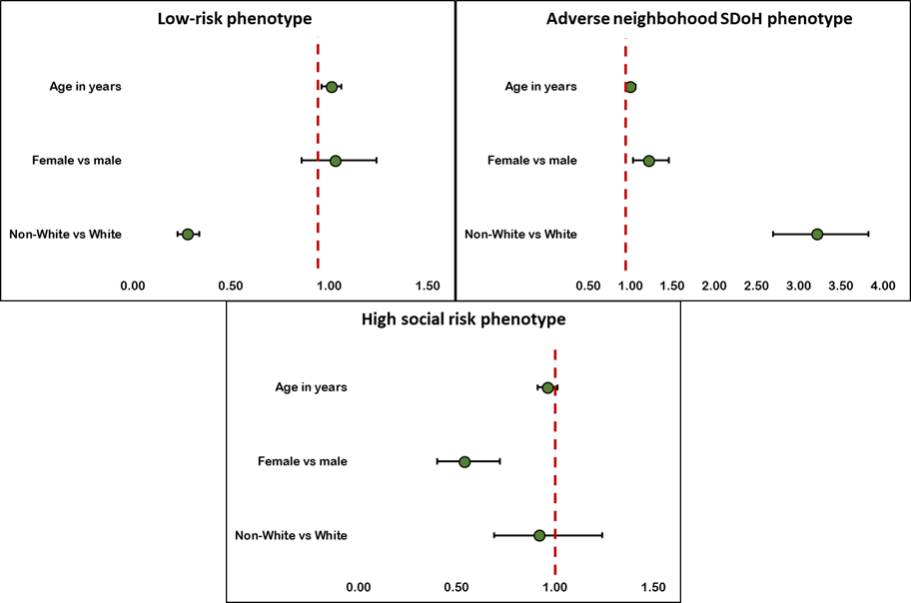
Prevalence odds ratio plots of the associations between age, gender, and race and phenotype status among patients identified in the electronic medical record in social risk data available in 2020. The points are prevalence odds ratio estimates and the lines are 95% CIs. SDoH: social determinants of health.

Upon examining whether age, gender, and race characteristics were associated with phenotype status, we found (Figure 4) that the adverse neighborhood SDoH phenotype was more prevalent among female and non-White patients (POR 1.22, 95% CI 1.03‐1.46 and POR 3.21, 95% CI 2.69‐3.82, respectively). The high social risk phenotype was more likely to be younger and male, while the low risk phenotype was more likely to be older and White.

**Table 3.** Characteristics by social determinants of health phenotype produced by latent class analysis models using patients identified in the electronic medical record with social risk data available in 2020.

			Low-risk phenotype (n=810), n (%)	Adverse neighborhood phenotype (n=1353), n (%)	High social risk phenotype (n=217), n (%)	*P* value
**Personal characteristics**						
	**Age (years)**					<.001
		<40	157 (19.4)	310 (22.9)	70 (32.2)	
		40‐60	288 (35.6)	562 (41.5)	123 (56.7)	
		≥60	365 (45)	481 (35.6)	24 (11.1)	
	**Gender**					<.001
		Male	339 (41.9)	520 (38.4)	116 (53.5)	
		Female	471 (58.1)	833 (61.6)	101 (46.5)	
	**Race**					<.001
		White	571 (70.5)	506 (37.4)	105 (48.4)	
		Black or other	239 (29.5)	847 (62.6)	112 (51.6)	
	**Veteran**					.02
		No	755 (93.2)	1288 (95.2)	212 (97.7)	
		Yes	55 (6.8)	65 (4.8)	5 (2.3)	
**Family and home**						
	**Housing status**					<.001
		I have housing	808 (99.8)	1342 (99.2)	149 (68.7)	
		I do not have housing	2 (0.2)	11 (0.8)	68 (31.3)	
	**Housing insecurity**					<.001
		Not worried about losing housing	779 (96.2)	1315 (97.2)	107 (49.3)	
		Worried about losing housing	31 (3.8)	38 (2.8)	110 (50.7)	
**Socioeconomic status**						
	**Education**					<.001
		More than high school	514 (63.5)	499 (36.9)	60 (27.6)	
		High school diploma/General Educational Development certificate	257 (31.7)	670 (49.5)	91 (41.9)	
		<High school degree	39 (4.8)	184 (13.6)	66 (30.4)	
	**Employment**					<.001
		Employed	253 (31.2)	339 (25.1)	20 (9.2)	
		Retired	384 (47.4)	536 (39.6)	77 (35.5)	
		Unemployed	173 (21.4)	478 (35.3)	120 (55.3)	
	**Insurance**					
		Private	510 (63)	609 (45)	43 (19.8)	<.001
		Public	218 (26.9)	470 (34.7)	86 (39.6)	
		Self-pay	82 (10.1)	274 (20.3)	88 (40.6)	
	**Lack of resources**					<.001
		None reported	648 (80)	971 (71.8)	51 (23.5)	
		Needed	162 (20)	382 (28.2)	166 (76.5)	
	**Transportation**					<.001
		No need	780 (96.3)	1264 (93.4)	91 (41.9)	
		Nonmedical need	16 (2)	41 (3)	97 (44.7)	
		Medical need	14 (1.7)	48 (3.5)	29 (13.4)	
**Social and emotional health**						
	**Stress**					<.001
		Not at all	217 (26.8)	355 (26.2)	2 (0.9)	
		A little bit	208 (25.7)	379 (28)	2 (0.9)	
		Quite a bit	115 (14.2)	174 (12.9)	35 (16.1)	
		Somewhat	187 (23.1)	324 (23.9)	26 (12)	
		Very much	83 (10.2)	121 (8.9)	152 (70)	
	**Social support**					<.001
		More than 5 times a week	458 (56.5)	692 (51.1)	52 (24)	
		3 to 5 times a week	261 (32.2)	508 (37.5)	35 (16.1)	
		1 or 2 times a week	78 (9.6)	113 (8.4)	55 (25.3)	
		Less than once a week	13 (1.6)	40 (3)	75 (34.6)	
**Personal safety and vulnerability**						
	**Safe where I live**					<.001
		Yes	787 (97.2)	1306 (96.5)	138 (63.6)	
		No	23 (2.8)	47 (3.5)	79 (36.4)	
	**Afraid of my partner**					<.001
		No	796 (98.3)	1339 (99)	183 (84.3)	
		Yes	14 (1.7)	14 (1)	34 (15.7)	
	**Incarcerated**					<.001
		No	802 (99)	1332 (98.4)	186 (85.7)	
		Yes	8 (1)	21 (1.6)	31 (14.3)	
**Neighborhood level**						
	**Social Vulnerability Index^a^ overall**					<.001
		Low	471 (58.1)	16 (1.2)	21 (9.7)	
		Moderate	339 (41.9)	308 (22.8)	67 (30.9)	
		High	0 (0)	1029 (76.1)	129 (59.4)	
	**Rurality**					<.001
		Metropolitan	768 (94.8)	1097 (81.1)	195 (89.9)	
		Micropolitan	41 (5.1)	132 (9.8)	15 (6.9)	
		Small town	1 (0.1)	82 (6.1)	5 (2.3)	
		Rural	0 (0)	42 (3.1)	2 (0.9)	
	**Yost socioeconomic status index^b^**					<.001
		High	271 (33.5)	0 (0)	8 (3.7)	
		Moderate	539 (66.5)	144 (10.6)	51 (23.5)	
		Low	0 (0)	1209 (89.4)	158 (72.8)	

a Higher values indicate more vulnerabilities in census tract of residence.

b Lower values indicate worse neighborhood-level socioeconomic status in census tract of residence.

**Table 4.** Prevalence ratios for cardiometabolic conditions by phenotype at time of PRAPARE (Protocol for Responding to and Assessing Patient Assets, Risks, and Experience) among patients identified in the electronic medical record with social risk data available in 2020.

			Events, n	Prevalence ratio (95% CI)
**Diabetes**				
	Low risk		279	Reference
	Adverse neighborhood		553	1.19 (1.06‐1.33)
	High social risk		62	0.83 (0.66‐1.04)
**Obesity^a^**				
	Low risk		68	Reference
	Adverse neighborhood versus low risk		118	1.07 (0.84‐1.36)
	High social risk versus low risk		34	0.82 (0.58‐1.15)
**Glycosylated hemoglobin ≥7.0 units^b^**				
	Low risk		86	Reference
	Adverse neighborhood versus low risk		168	1.14 (0.92‐1.43)
	High social risk versus low risk		23	1.18 (0.80‐1.73)
**Other cardiometabolic conditions**				
	**Hypertension**			
		Low risk	306	Reference
		Adverse neighborhood versus low risk	582	1.14 (1.02‐1.27)
		High social risk versus low risk	72	0.88 (0.71‐1.08)
	**Coronary artery disease**			
		Low risk	190	Reference
		Adverse neighborhood versus low risk	343	1.08 (0.93‐1.26)
		High social risk versus low risk	47	0.92 (0.69‐1.22)
	**Myocardial infarction**			
		Low risk	120	Reference
		Adverse neighborhood versus low risk	218	1.09 (0.88‐1.33)
		High social risk versus low risk	37	1.15 (0.82‐1.61)
	**Peripheral vascular disease**			
		Low risk	56	Reference
		Adverse neighborhood versus low risk	137	1.46 (1.09‐1.97)
		High social risk versus low risk	15	0.99 (0.57‐1.73)
	**Cardiomyopathy**			
		Low risk	43	Reference
		Adverse neighborhood versus low risk	97	1.35 (0.95‐1.91)
		High social risk versus low risk	18	1.56 (0.92‐2.65)
	**Heart failure**			
		Low risk	115	Reference
		Adverse neighborhood versus low risk	282	1.46 (1.20‐1.79)
		High social risk versus low risk	38	1.23 (0.88‐1.72)

a n=610.

b n=945.

## Discussion

In this LCA of over 2300 patients who completed a standardized social risk screener in the electronic medical record at a large academic institution, we found 3 distinct SDoH phenotypes—high social risk, adverse neighborhood, and low social risk. Patients with an adverse-neighborhood SDoH phenotype—characterized by living in census tracts with high social vulnerability, poor neighborhood socioeconomics, and rural or small town locales—were more likely to have a diagnosis of T2D and other cardiovascular-related conditions. Although not significant, we did find increased prevalence for uncontrolled HbA_1c_ among both the high-social-risk and adverse-neighborhood SDoH phenotypes. Although preliminary in nature, our findings suggest that combinations of individual- and neighborhood-level SDoH clusters in patient populations result in distinct phenotypes with divergent cardiometabolic outcomes. Our findings complement other investigations that have found distinct clusters in clinical cardiometabolic factors [34,35], SDoH in general [29,30], and SDoH clusters in women with HIV [21,22], as well as data reduction work using SDoH to create composite domains that were associated with cognition and health-related quality of life in older adults [52].

We found that the prevalence of patients with diabetes, hypertension, heart failure, and peripheral vascular disease was higher among those in the adverse-neighborhood SDoH phenotype compared to those in the low-risk phenotype. These findings are consistent with the existing literature describing associations between living in underresourced neighborhood environments and cardiometabolic disease prevalence and incidence [2,3,53]. Here, we found that these patients clustered together based on area-level characteristics despite incorporating information on individual social risks, suggesting that neighborhood environment plays a role even if individual measures of SES are not suboptimal per se. While the adverse-neighborhood SDoH phenotype did report adverse individual-level SDoH on the PRAPARE, these indicators did not account for the divergence of this phenotype from other phenotypes in our data. These findings contrast with recent findings from the All of Us study [29], which found phenotypes with a mixture of neighborhood characteristics and individual factors (eg, one phenotype included neighborhood characteristics, health insurance status, and social isolation status).

When we examined if age, gender, or race predicted belonging to any of the phenotypes, we found that those in the adverse-neighborhood SDoH phenotype were more likely to be non-White and female. Extensive literature has associated both female gender and non-White race with increased odds of cardiometabolic outcomes and complications. Our findings suggest—not surprisingly—that an interplay of gender, race, and adverse neighborhood socioeconomic conditions is associated with cardiometabolic outcomes, highlighting the need to identify subpopulations such as these for targeted intervention. While our results need further refinement and validation, they suggest that identifying female, non-White individuals that live in underresourced neighborhoods for cardiometabolic disease prevention and control would be a high priority in our hospital catchment.

Interestingly, we did not find a higher prevalence of cardiometabolic conditions among the high-social-risk phenotype compared to the low-risk phenotype. The higher level of stress in this phenotype was also interesting, and it was perhaps associated with substance use and other behaviors, warranting future exploration. Our lack of cardiometabolic association findings in this group may be due to reverse causality, where estimations may be confounded due to underlying disease [54]. Similarly, this group may have had fewer encounters with the health system and thus fewer opportunities to be diagnosed. Moreover, this phenotype was, on average, younger than the other phenotypes; thus, development of chronic conditions may have not been captured in the life course yet. We did find that patients in the high-social-risk phenotype had an increase in uncontrolled HbA_1c_ at the time of the PRAPARE assessment among a subset who had lab values available (n=945), although this was not statistically significant. This concerning finding warrants further exploration among a larger sample since, if this finding holds, these patients carry a high burden of social risk coupled with undiagnosed cardiometabolic disease and represent a vulnerable population that should be identified and prioritized at the medical encounter.

Our SDoH phenotype findings are notable in terms of clinical utility and population health initiatives. It is estimated that SDoH account for 30%‐55% of an individual’s clinical outcomes [55], highlighting the necessity of acknowledging these factors in the context of medical care and population health promotion [56]. In fact, there have been recent calls for routine collection of SDoH at the medical encounter to facilitate early diagnosis, risk stratification, prevention efforts, and clinical care improvement in the midst of a growing global cardiometabolic disease burden [57]. The SDoH phenotypes found in our investigation provide valuable information on subpopulations that may benefit from targeted interventions, public health initiatives, or clinical care—in general as well as in connection to cardiometabolic disease. There are few studies to date that have attempted to cluster SDoH, with little guidance thus far on how to match interventions to clusters. Our group is currently conducting qualitative work examining how SDoH clusters found in our analysis map to potential interventions, tailoring of interventions, or social referrals to meet needs. Lastly, we used data accessible in the EMR via the PRAPARE and public data sources linked to geocoded patient addresses, underscoring the potential utility of using these phenotypes at the point of care with real-time data to facilitate risk prediction and risk stratification [58].

Our study has several strengths. To our knowledge, we are the first to use data collected via a standardized social risk screener in the EMR and area-level public data to detect SDoH phenotypes among a patient population and investigate associations between phenotype status and cardiometabolic-related disease. Moreover, we found preliminary evidence that suggests that patients living in adverse neighborhoods, regardless of their individual level of SDoH, may be a specific subpopulation at increased risk for diabetes. This also suggests that identifying and targeting certain vulnerable neighborhoods or geographies for outreach or community-level interventions to help reduce cardiometabolic disease incidence are warranted, as has been advocated in infectious disease work [59]. Further, our work lays the foundation for future work to examine if these phenotypes can be replicated in larger samples and various patient populations as well as qualitative investigations that query patients with specific phenotypes regarding their individual SDoH and personal health outcomes.

Our study is not without limitations. First, our study population consisted of patients administered the PRAPARE during an initial implementation period in the health system, resulting in a convenience sample that is not necessarily representative of the health care system as a whole and certainly not generalizable to populations outside of our local catchment and geographic region. Further, the PRAPARE was administered during the COVID-19 pandemic, and our previous report noted differences in SDoH prevalence before and after the pandemic, which should be noted here [18]. Second, the PRAPARE was administered at clinical encounters and may have excluded patients with adverse SDoH, resulting in selection bias. Third, we used LCA to detect phenotypes in our data; sophisticated methods such as segmentation and artificial intelligence modeling may provide more information regarding true phenotypes. Fourth, we had a high level of missing PRAPARE and area-level data, which may have also biased our estimates. We examined the missing data and found that they were more likely to come from younger or male individuals. Interestingly, these individuals may constitute their own phenotype and should be examined in linkage to health outcomes in future investigations. Our estimates reported here may be underestimates due to lack of information from these individuals. Lastly, we did not have BMI and HbA_1c_ values for the full data set, which would have been valuable to help detect particularly vulnerable patient populations with high social risk and undiagnosed diabetes.

In sum, we found that SDoH phenotypes were detectable in our patient sample and related to health outcomes in divergent ways. Our findings have implications for both clinical and research-related work in several ways. First, these phenotypes account for multiple correlated SDoH and can be linked to outcomes to better understand treatment or intervention effects. Second, clinicians can use such phenotypes to identify those at highest risk to prescribe appropriate treatments. Third, our results suggest that if elevated T2D and cardiometabolic prevalence are strongly associated at the neighborhood level, independent of individual-level characteristics, interventions should prioritize community engagement efforts in neighborhoods with adverse SDoH characteristics using a multilevel approach, from individual to community to policy. Future work should seek to validate these findings, as well as work to match interventions to phenotype group characteristics—be they social or clinical in nature.

## Supplementary material

10.2196/53371 Multimedia Appendix 1 Description of neighborhood-level measures.

## Abbreviations

aBIC: adjusted Bayesian information criterion
AIC: Akaike information criterion
BIC: Bayesian information criterion
cAIC: consistent Akaike information criterion
EMR: electronic medical record
HbA_1c_: glycosylated hemoglobin
*ICD-10*: *International Classification of Diseases, Tenth Revision*
IRB: institutional review board
LCA: latent class analysis
nSES: neighborhood SES index
POR: prevalence odds ratio
PR: prevalence ratio
PRAPARE: Protocol for Responding to and Assessing Patient Assets, Risks, and Experience
RUCA: rural-urban commuting area
SDoH: social determinants of health
SEER: Surveillance, Epidemiology, and End Results
SES: socioeconomic status
SVI: Social Vulnerability Index
T2D: type 2 diabetes
UAB: University of Alabama at Birmingham
