# Supplementary material for: Social Determinants of Health Phenotypes and Cardiometabolic Condition Prevalence Among Patients in a Large Academic Health System: Latent Class Analysis
Source: JMIR Public Health Surveill. 2024 Aug 7;10:e53371. doi: 10.2196/53371 (PMC11322797; doi:10.2196/53371)
Supplement: Multimedia Appendix 1 [file publichealth-v10-e53371-s001.docx]

**Supplemental Material**

Social Vulnerability Index (SVI): The SVI is a composite index using census data indicators on social factors, used to describe the social conditions that may influence human suffering and financial hardship (social vulnerabilities) for disaster planning. The index is available by census tract and vulnerability has been linked to poor health outcomes. The SVI from 2018 used 15 census tract indicators grouped into 4 themes. Complete data documentation can be found here: <https://www.atsdr.cdc.gov/placeandhealth/svi/data_documentation_download.html>

| Theme | Census Data |
| --- | --- |
| SES Status | % below poverty |
|  | % unemployed |
|  | Income |
|  | No high school diploma |
| Household Composition | % Aged 65 and older |
|  | % Aged 17 and younger |
|  | % Civilian with a disability |
|  | Single-Parent households |
| Minority Status and Language | % Minority population |
|  | % aged 5 and older who speak English “less than well” |
| Housing and Transportation | Multi-unit structures |
|  | Mobile Homes |
|  | Crowding |
|  | No vehicle |
|  | Group Quarters |

Yost SES Index^40^: The Yost SES Index is a time-dependent composite score which utilizes seven components from census data linked to the census tract level: education index^41^, percent persons living 200% below poverty line, median household income, median house value, median rent, percent blue collar workers, and percent older than 16 in the workforce without a job. Indices were estimated using the American Community Survey (ACS) 5-year estimates. Principal component analysis was used to develop a weighted linear combination of variables from the data to create a value for each census tract.
